# Effects of agricultural pesticides on the susceptibility and fitness of malaria vectors in rural south-eastern Tanzania

**DOI:** 10.1186/s13071-022-05318-3

**Published:** 2022-06-16

**Authors:** Naomi H. Urio, Polius G. Pinda, Amos J. Ngonzi, Letus L. Muyaga, Betwel J. Msugupakulya, Marceline Finda, Godfrey S. Matanila, Winifrida Mponzi, Halfan S. Ngowo, Najat F. Kahamba, Theresia E. Nkya, Fredros O. Okumu

**Affiliations:** 1 Environmental Health and Ecological Science Department, Ifakara Health Institute, P.O. Box 53, Ifakara, Tanzania; 2 School of Life Science and Bioengineering, The Nelson Mandela African Institute of Science and Technology, P.O. Box 447, Arusha, Tanzania; 3 School of Public Health, Faculty of Health Sciences, University of the Witwatersrand, Johannesburg, South Africa; 4 Department of Vector Biology, Liverpool School of Tropical Medicine, Liverpool, UK; 5 International Centre of Insect Physiology and Ecology, Nairobi, Kenya; 6 University of Dar es Salaam, Mbeya Health and Allied Sciences, Mbeya, Tanzania; 7 Institute of Biodiversity, Animal Health, and Comparative Medicine, University of Glasgow, G12 8QQ Glasgow, Scotland

**Keywords:** *Anopheles arabiensis*, Insecticide susceptibility/resistance, Agricultural pesticides, Fecundity, Malaria, Focus group discussion, Ifakara Health Institute

## Abstract

**Background:**

Agricultural pesticides may exert strong selection pressures on malaria vectors during the aquatic life stages and may contribute to resistance in adult mosquitoes. This could reduce the performance of key vector control interventions such as indoor-residual spraying and insecticide-treated nets. The aim of this study was to investigate effects of agrochemicals on susceptibility and fitness of the malaria vectors across farming areas in Tanzania.

**Methods:**

An exploratory mixed-methods study was conducted to assess pesticide use in four villages (V1–V4) in south-eastern Tanzania. *Anopheles* *gambiae* (s.l.) larvae were collected from agricultural fields in the same villages and their emergent adults examined for insecticide susceptibility, egg-laying and wing lengths (as proxy for body size). These tests were repeated using two groups of laboratory-reared *An. arabiensis*, one of which was pre-exposed for 48 h to sub-lethal aquatic doses of agricultural pesticides found in the villages.

**Results:**

Farmers lacked awareness about the linkages between the public health and agriculture sectors but were interested in being more informed. Agrochemical usage was reported as extensive in V1, V2 and V3 but minimal in V4. Similarly, mosquitoes from V1 to V3 but not V4 were resistant to pyrethroids and either pirimiphos-methyl or bendiocarb, or both. Adding the synergist piperonyl butoxide restored potency of the pyrethroids. Pre-exposure of laboratory-reared mosquitoes to pesticides during aquatic stages did not affect insecticide susceptibility in emergent adults of the same filial generation. There was also no effect on fecundity, except after pre-exposure to organophosphates, which were associated with fewer eggs and smaller mosquitoes. Wild mosquitoes were smaller than laboratory-reared ones, but fecundity was similar.

**Conclusions:**

Safeguarding the potential of insecticide-based interventions requires improved understanding of how agricultural pesticides influence important life cycle processes and transmission potential of mosquito vectors. In this study, susceptibility of mosquitoes to public health insecticides was lower in villages reporting frequent use of pesticides compared to villages with little or no pesticide use. Variations in the fitness parameters, fecundity and wing length marginally reflected the differences in exposure to agrochemicals and should be investigated further. Pesticide use may exert additional life cycle constraints on mosquito vectors, but this likely occurs after multi-generational exposures.

**Graphical Abstract:**

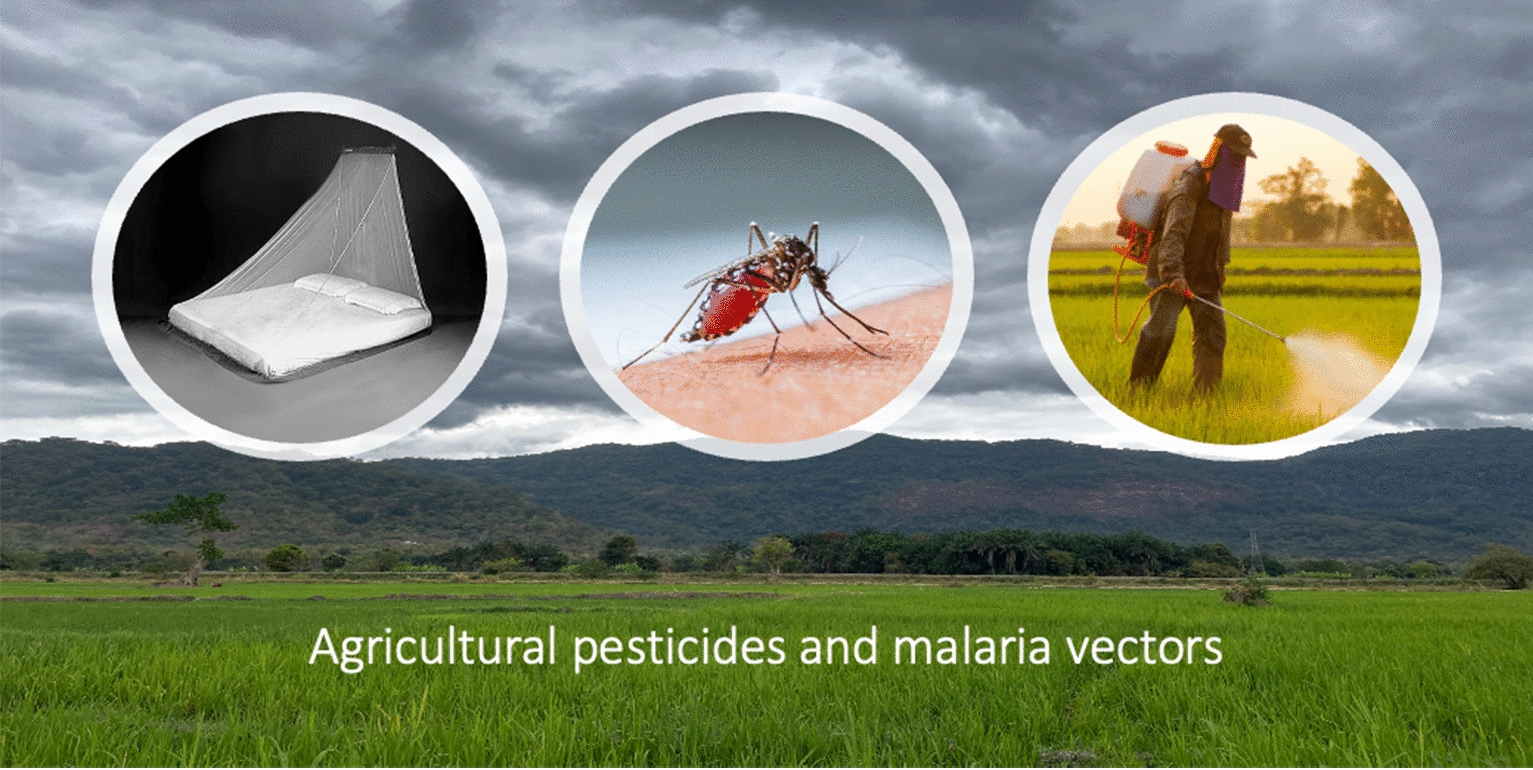

## Background

Vector control has played a key role in the control of malaria globally [1–5]. The primary interventions, namely insecticide-treated nets (ITNs) and indoor residual spraying (IRS), are estimated to have contributed nearly 70% of all the gains accrued against malaria starting in 2000 [1]. Unfortunately, the disease remains a major public health concern in sub-Saharan Africa, where over 95% of the cases and deaths now occur [1, 6]. Despite the gradual declines that started around 2000, the incidence and mortality rates of malaria now appear to be increasing, especially in the high-burden countries [6]. The success of vector control interventions is now threatened by multiple challenges, including, but not limited to, certain shifts in malaria vector species populations [7, 8], changes in mosquito biting patterns [9, 10] and the widespread insecticide resistance in mosquito populations [11]. Insecticide resistance is particularly challenging because the chemicals are widely used in both public health and agriculture, yet the stewardship of these products is not integrated.

The broad linkages between agricultural practices and transmission of insect-borne diseases are widely appreciated in Africa and elsewhere [12–17]. On one hand, crop farming and livestock keeping are an important basis of livelihoods for millions of people in malaria-endemic communities [18, 19]. On the other, vector-borne and zoonotic diseases can disrupt the same livelihoods by lowering productivity and draining household incomes [13, 20]. While the resulting impacts of specific farming practices are varied [21], evidence suggests multiple associations between the ecology of malaria vectors and cultivation of crops such as rice and sugarcane [12, 21–24]. Suitable ecological conditions, such as the shallow and slow-moving fresh water systems found in rice fields, may contribute to higher densities of malaria vectors compared to non-rice-growing areas [25–27]. Large populations of vector species such as *Anopheles arabiensis* may proliferate in rice-growing areas and can increase malaria transmission in these farming regions [27–29].Other forms of agriculture, i.e. livestock keeping, may also influence malaria transmission. For example, mosquito blood-feeding is influenced by the availability and densities of non-human vertebrates and livestock [30, 31].

The use of agricultural pesticides is perhaps the most adversely linked to malaria transmission. Resistance in malaria vectors is predominantly attributed to selection pressures when mosquitoes are exposed to public health insecticides, mainly IRS and ITNs [32, 33]. However, agricultural pesticides potentially also contribute to resistance through the selection pressures imparted during the aquatic stages [34–36], as mosquitoes emerging from areas of intense pesticide use show decreased susceptibility to insecticides [34, 35, 37, 38]. For example, in Côte d’Ivoire, extensive use of pesticides was associated with significant loss of susceptibility to key malaria vector species [39]. This association between agriculture and malaria control is strongly attributed to similarities in chemicals used, modes of action, simultaneous application of these chemicals and their extensive use in agriculture [16, 40].

Since most investigations in this subject have been aimed at safeguarding the performance of insecticide-based vector control tools, notably ITNs and IRS, there is limited understanding of how agrochemicals actually influence malaria vector populations or their fitness and survival parameters. Similarly, whereas it is generally agreed that insecticide-resistant mosquitoes incur significant fitness costs compared to susceptible mosquitoes, it remains unclear how such factors influence the overall pathogen transmission potential. A recent review concluded that while resistance-associated fitness costs are common, the available evidence is difficult to summarise because of the variations in resistance mechanisms, different insecticides tested and inconsistencies in experimental designs [41].

Insecticide resistance is broadly an energy-intensive process, which leads to changes in mosquito physiology and behaviours and might affect the vector competence [42], usually in the context of other biological factors that influence vectorial capacity either favourably or negatively [43–45]. These biological characteristics often include larval development, survival and fecundity, all of which can be altered in insecticide-resistant vector populations [46]. In a study from Cameroon, researchers established a colony of field-collected *Anopheles* mosquitoes strongly resistant to both DDT and pyrethroids and then assessed different life traits including fecundity, larval development, emergence rates and longevity of surviving adults. Compared to mosquitoes from a susceptible laboratory colony, they observed that the resistant *Anopheles* had fewer eggs, delayed larval development, reduced emergence rates and reduced survival of the emergent adults [47]. Another study in Kenya showed that pyrethroid-resistant *An. gambiae* had delayed development during aquatic stages and also reduced survivorship [48]. Furthermore, malaria infection rates in West Africa were higher in resistant mosquitoes than in susceptible ones, even though the latter had lower proportions of parous females [49]. Studies on *Aedes* have demonstrated similar fitness costs, sometimes extending to reduced mating success [50], though in some cases these costs were marginal [51]. Taken together, these findings suggest that the associations between resistance and the fitness of vectors are complex and should be investigated for different localities to examine how exposures to specific pesticides may influence overall fitness and the resulting malaria transmission.

In rural south-eastern Tanzania, Matowo et al. found that insecticides used in agriculture and vector control share similar active ingredients, and many farmers in the area had poor pesticide management and awareness [16]. In these areas, the malaria vector species *An. arabiensis* is known to breed in the same fields where pesticides are used. These vector populations are therefore endlessly exposed to insecticides either in the aquatic stage on the farms or when the adults come into contact with insecticide-impregnated bed nets inside homes, thus increasing resistance pressures. Entomological surveys have demonstrated that the insecticide resistance in the vectors here varies with season and location [52, 53] and is much stronger in *An. funestus* than in *An. arabiensis* [53]. It is plausible that the resistance in the area may be at least partly linked to agricultural pesticide use patterns and is contributing to the persistent transmission by the residual malaria vectors. However, there is insufficient knowledge on how pesticide use may be directly or indirectly influencing the fitness and transmission activity of the dominant malaria vectors.

This study was therefore aimed at investigating the effect of agrochemicals on the insecticide susceptibility status of *An. arabiensis* and how that affects the fitness parameters on pesticide pre-exposed *An. arabiensis* in rural south-eastern Tanzania.

## Methods

### Study site

**Fig. 1:**
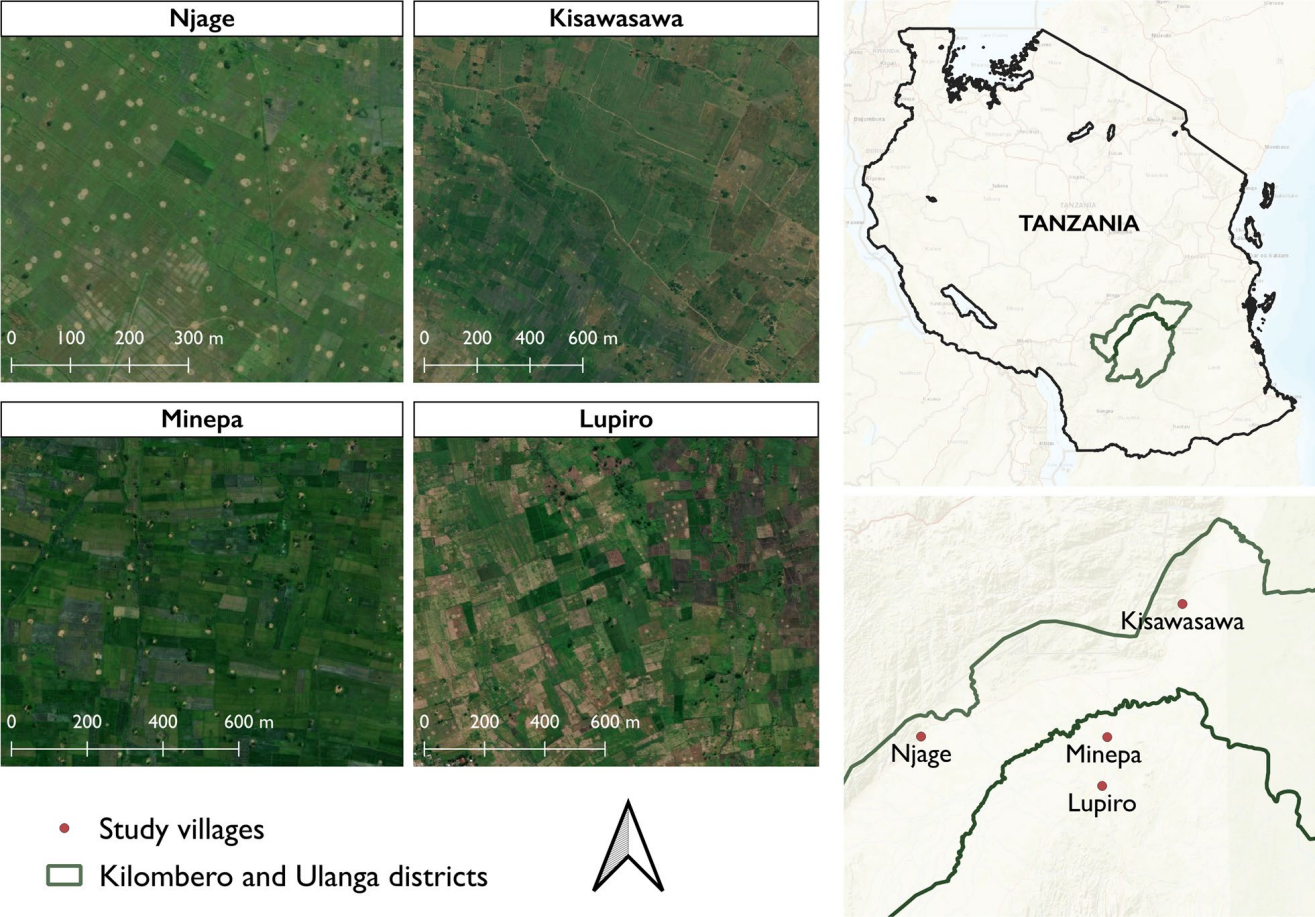
Map of Kilombero and Ulanga districts, Tanzania, showing the villages where the study was conducted

The field study was conducted in agricultural districts of Ulanga and Kilombero in south-eastern Tanzania, where the main malaria vectors include *Anopheles funestus* and *Anopheles arabiensis* [52, 54]. Four villages were covered, namely Minepa (V1) 8.23°S, 36.75°E; altitude 268 m) and Lupiro (V2) (8.41°S, 36.81°E; altitude = 389 m) in Ulanga district and Kisawasawa (V3) (7.91°S, 36.82°E; altitude = 728 m) and Njage (V4) (8.3°S, 36.14°E; altitude = 519 m) in Kilombero district (Fig. 1). The area experiences 15–35 °C daily temperatures and 1300–3600 mm annual precipitation [55]. Economic activities include crop farming, fishing and livestock keeping [16, 56]. The main malaria vector control method in the area is ITNs [57].

### Qualitative assessment of the use of agricultural pesticides

A qualitative assessment was conducted in the four study villages by carrying out focus group discussions (FGDs) to identify the common agricultural pesticides used in each village, followed by direct observations of pesticide use in the farms.

A discussion guide was prepared for the FGDs to capture the following key areas of discussion: (i) farming practices, (ii) types of chemicals used on the farm, (iii) farming seasons and crop types, (iv) sources of agricultural pesticides and (v) management and disposal of the pesticides. An FGD was conducted in each village, bringing together eight local community representatives recruited upon signing the informed consent. These representatives were community leaders, practicing subsistence farming, and included both male and female adults. The discussions were facilitated by three researchers from Ifakara Health Institute, who were all knowledgeable about agricultural practices and malaria control interventions in the region and had experience conducting qualitative research in rural communities. Before starting the discussions, facilitators presented a brief overview of the study topic and the reason for conducting the FGDs. Each session lasted about 60–75 min.

In addition, direct observations were made in the farms in the study villages to record signs of pesticide use, disposal and methods of application. Where possible, pictorial evidence was gathered to complement the direct observations.

### Mosquito collections and rearing of larvae

Field mosquitoes were collected in their larval stages during the dry season months of July and September 2021. The study villages were inspected for presence of aquatic habitats known to contain *An. gambiae* (s.l.) mosquitoes before the actual mosquito collections were done. Standard 350 ml dippers were used to collect the larvae, and the collections were transported to the VectorSphere Mosquito Laboratory facility at Ifakara Health Institute for further experiments. In the insectary, the larvae were kept in  5L rearing dishes under controlled conditions of 25–27 °C and 80% relative humidity. The larvae were fed on Tetramin^®^ fish food, and the emergent adults were supplied with a 10% sugar solution. As described below, An. *arabiensis* females from a laboratory colony maintained under the same conditions since 2009 were also used in some comparative tests.

### Assessment of insecticide susceptibility of the field-collected mosquitoes

Emergent adult female mosquitoes aged 3–5 days old and not previously blood-fed were used according to the WHO insecticide susceptibility test procedures [58]. A total of 120 mosquitoes per test were used over four replicates, each requiring 20–25 mosquitoes. The bioassays were done at 25 °C ± 2 °C and 80% ± 10% relative humidity.

The candidate insecticides and doses included: 0.75% permethrin (pyrethroids class I), 0.05% lambda-cyhalothrin (pyrethroid class II), 0.25% pirimiphos-methyl (organophosphate), 4% DDT (organochloride) and 0.1% bendiocarb (carbamate). The mosquitoes were exposed for 1 h and knockdown recorded at intervals of 10, 15, 20, 30, 40, 50 and 60 min. The mosquitoes were then transferred to clean holding tubes, supplied with 10% sugar solution and observed to assess mortality after 24 h. For comparison, similar tests were conducted using the laboratory-reared mosquitoes.

A synergist, 4% piperonyl butoxide (PBO), was used to assess possible involvement of mixed-function oxidases in metabolic resistance [58]. In these tests, the mosquitoes were pre-exposed to PBO or control and then to the candidate insecticides. Four groups of 20–25 mosquitoes each were used, and the groups were exposed for 1 h to PBO alone, PBO followed by respective candidate insecticide for 1 h, the candidate insecticide alone or the control papers. The mosquitoes were monitored for 24-h mortality and the tests replicated three times.

### Assessment of fitness parameters of field-collected mosquitoes

Field-collected larvae from the four study villages (V1–V4) that were brought to the insectary and kept till they pupated, after which they were collected in cups and placed in standard 15 × 15 × 15-cm cages. Upon adult emergence, the mosquitoes were fed on 10% glucose solution via soaked cotton pads. Mosquitoes were provided their first blood meal on the 3rd day after emergence to give them time to mate. Fully fed mosquitoes were moved into individual cups with a damp filter paper at the base of the cup to stimulate oviposition conditions. Eggs laid by each mosquito were counted under a stereomicroscope as a measure of fecundity.

Wing sizes were also measured and used as a proxy for body size. Adult female mosquitoes emerging from the field larva collections were anaesthetized at −10 °C for 7 min, and a single wing was removed from either the left or right side of the mosquito. Distilled water was used to fix the wings onto the slides, and the length from the apical notch to the auxiliary margins was measured using a micrometer ruler under a stereo-microscope.

### Exposure of mosquitoes to sub-lethal doses of agricultural pesticides

These tests were done to simulate field exposures to common agrochemicals and assess effects on emergent adults. Groups of third instar *An. arabiensis* larvae from the laboratory colonies were exposed to a range of pesticide concentrations (Table 1) to observe the effect of the active treatments and determine the doses below which there were still substantial emergent adults for further experimentation. For this experiment, three insecticides from three pesticide classes (pyrethroids, organophosphates and carbamates) were selected to be representative of the most common pesticide classes used by communities in the study villages (as determined by the FGDs and field observations).

**Table 1:** Selected pesticides used for experiments of sub-lethal exposures of *Anopheles* mosquitoes

Trade name	Insecticide class	Active ingredient	Concentration of active ingredient	Description and recommended use
Ninja 5EC	Pyrethroid	Lambda-cyhalothrin	50 g/l	Fast-acting and broad-spectrum; for use on cashew nuts, vegetables and fruits; targets sucking insect pests
Twigaphos 48EC	Organophosphate	Chlorpyrifos	480 g/l	For the control of insects in cotton, coffee, cashew and vegetables
Akheri Powder	Carbamate	Carbaryl	5% w/w	For domestic use against crawling insects such as fleas, ants, cockroaches, etc.

The pesticides represent the different insecticide classes used in the study villages

In each test, 25 larvae were introduced into 1.2L basins containing 1L of water, into which the candidate pesticides were introduced. The pesticide concentrations ranged from 1 × 10^−4^ to 1 × 10^−8^ g/l (for lambda cyhalothrin and pyrimiphos methyl) and 5 × 10^−4^ to 3.5 × 10^−3^ g/l (bendiocarb). Three replicates were completed for each concentration (treatment) and control, all under the same conditions. All larvae, exposed and unexposed, were fed on Tetramin^®^ fish food and monitored every 12 h for mortality until 120 h.

A lethal concentration (LC) of 15% mortality was selected for subsequent experiments, since at this level there was sufficient adult emergence despite significant pesticide exposure. Fresh batches of fourth-instar larvae were therefore exposed to the LC_15_ pesticide concentrations in 1.2L trays for 48 h, during which the larvae were fed Tetramin® fish food. Pupae from these trays were transferred to cups containing only water and placed in small cages (15 × 15 × 15 cm) for emergence.

### Assessment of insecticide susceptibility and fitness parameters of the laboratory mosquitoes

Once the sub-lethal dose had been determined, the third to fourth instar larvae were introduced and maintained until pupation. The pupae collected from each treated dish (exposed mosquitoes) and from concurrent controls (unexposed mosquitoes) were counted and placed in cages for emergence. Three-to-five-day-old female adult mosquitoes were then used to carry out insecticide susceptibility bioassays as described above for field-collected mosquitoes. Fecundity and wing length were also assessed in the same way as described above for field-collected mosquitoes.

### Data analysis

The quantitative data were analysed using the open-source software, R programming version 4.0.5 [59]. In tests using mosquitoes pre-exposed to sub-lethal aquatic doses of pesticides, Probit analysis was done using the ‘*ecotox*’ library [60] to determine appropriate sub-lethal concentrations to be used in subsequent experiments. To assess the insecticide resistance profiles, data analysis was done according to the WHO susceptibility test guidelines. The resistance or susceptibility status was defined based on the WHO criteria, where 98–100% mortality indicates susceptibility; 90–97% mortality requires further confirmation of possible resistance, and < 90% mortality indicates resistance [58]. The resistance/susceptibility graphs were plotted using *ggplot2* package [61]. Knockdown times were calculated using the PoLo Plus software [62] using log-probit analysis. For fecundity and wing length, the *dabestr* package in R was used to generate two-group estimation (Gardner-Altman) plots [63]. The estimation plots were used to display distributions of residual mean differences in the number of eggs laid and wing lengths in the different experiments. The results are presented in summary graphs or tables.

For the qualitative assessment, audio recordings of the focus group discussions and key informant interviews were transcribed and translated from Swahili to English. Any notes written during the discussion were added to the written transcripts. The written transcripts were reviewed and analysed on Microsoft Word. Analyses were conducted separately for different villages. The FGD guide and objective of the study were used to develop deductive codes. Inductive codes were derived from detailed studying of the written transcripts. Once the coding was completed, codes were grouped, and emerging patterns were used to identify themes.

## Results

### Farming practices in south-eastern Tanzania

Rice farming was the most common across the four villages and was either rain or irrigation dependent. Most villages reported cultivating rice during both seasons. During the wet season they depended on rain as a source of water, and during the dry season they relied mostly on irrigation systems with farms near rivers to access the water. Their planting methods were broadcasting seeds, planting single seeds or transplanting seeds. A common challenge was the need to spray agrochemicals on the farms at the same time to avoid re-invasion of pests from neighbouring farms. The participants noted that in cases where this was not done, they often had to re-spray because of such re-invasions:

“Spraying every two weeks is not fixed, you can spray pesticides every week. For example, when having two adjacent plots, the owner of one’s crops has been attacked by pests because they cannot afford to purchase pesticides. If the other plot owner decides to spray pesticides on his own land, pests will die but after a few days, the pesticide has worn out and pests from the adjacent plot will attack again. So, you find yourself spraying pesticides multiple times every week. Unless you decide to buy and spray your neighbour’s plot with pesticides if you can afford to.” (Male, 45 years)

### Common herbicides and pesticides used

The use of herbicides was common across villages. Farmers reported mostly using bispyribac-sodium- and glyphosate-containing herbicides to control weeds on their farms. Participants from three out of four of the villages (i.e. Villages V1, V2 and V3) reported relying on pesticides to deal with pests affecting their crops. The most common pesticides reported were of pyrethroid and organophosphate classes (Table 2). Herbicides and pesticides were used multiple times because of the frequent occurrence of weeds and pests on their crops. It was also mentioned that application of these chemicals was greater during the dry seasons compared to wet seasons because of the abundance of pests and weeds in that period. Most farmers in these villages disposed of their pesticide containers poorly by leaving them in either their fields or nearby rivers. However, farmers in one village (V4) reported barely using pesticides on their farms, noting that they did not find it necessary:

“When we talk about pests affecting our crops, I can say that about 97% of farmers in our village are not really affected by pests, as it is not too big of a problem. To say that we look for alternative methods to look for pesticides that work is rare, we take it as a normal thing, which does not have major effects on us as farmers.” (Male, 31 years)

**Table 2:** Common agricultural pesticides used in respective villages, their chemical class, frequency of use and resistance status

Village	Pesticide/herbicide	Trade name	Chemical class	Active ingredient	Frequency of use	Resistance status
V1	Both pesticides & herbicide (widespread use)	Karate 5EC	Pyrethroid	Lambda-cyhalothrin	High	CR
		KungFu 5EC	Pyrethroid	Lambda-cyhalothrin	High	CR
		Rapid Attack 344SE	Pyrethroid + Neonicotinoids	Cypermethrin + Imidacloprid	Moderate	CR−
V2	Both pesticides & herbicide (widespread use)	KungFu 5EC	Pyrethroid	Lambda-cyhalothrin	High	CR
		Profecron 720EC	Organophosphate	Profenofos	Moderate	PR
		Dasba 40EC	Organophosphate	Chlorpyrifos	Moderate	PR
V3	Both pesticides & herbicide(widespread use)	Actellic 50EC	Organophosphate	Pirimiphos-methyl	High	PR
		Karate 5EC	Pyrethroid	Lambda-cyhalothrin	High	CR
		KungFu 5EC	Pyrethroid	Lambda-cyhalothrin	High	CR
		Profecron 720EC	Organophosphate	Profenofos	High	CR
V4	Only herbicides (marginal use)	2, 4 D Amine	Aryloxyacides II	2, 4 D- dimethyl amine salt	Moderate	–

V1 = Minepa, V2 = Lupiro, V3 = Kisawasawa, V4 = Njage

*CR* confirmed resistance, *PR* possible resistance

### Awareness of the linkages between agricultural pesticide use and insecticide resistance and mosquito control

Many of the community leaders across all the farming villages were not aware of the association between the chemicals used on their farms and malaria vector control such as ITNs and IRS. There was no evidence of the farmers associating agricultural pesticide use with insecticide resistance in mosquitoes. However, there was an association of pesticide use with mosquito mortality. In one village (V2), the farmers reported that they knew that the mosquito larvae died immediately after they had sprayed chemicals on their farms. However, they had never tried to use the same chemicals to kill mosquitoes at home or surrounding areas:

“We know when we have killed mosquitoes and the scientists don’t get anything when they come and collect mosquitoes on the rice farms.” (Male, 60 years)

Farmers in all four villages mentioned that they wanted to know more about the association between agrochemical use on their farms and malaria control. They were willing to undergo any form of training to improve their knowledge on the subject at hand. They suggested that experts in the respective sectors should advise farmers on which chemicals to use in order not to harm malaria control efforts but still maintain their farm produce

“We can sit together with scientists and listen to advise given to us on the types of chemicals to use so that we do not affect malaria control. We can make plans that can help get rid of some barriers in malaria control efforts.” (Female, 49 years)

### Susceptibility and fitness parameters of field-collected mosquitoes

#### Insecticide susceptibility

**Fig. 2:**
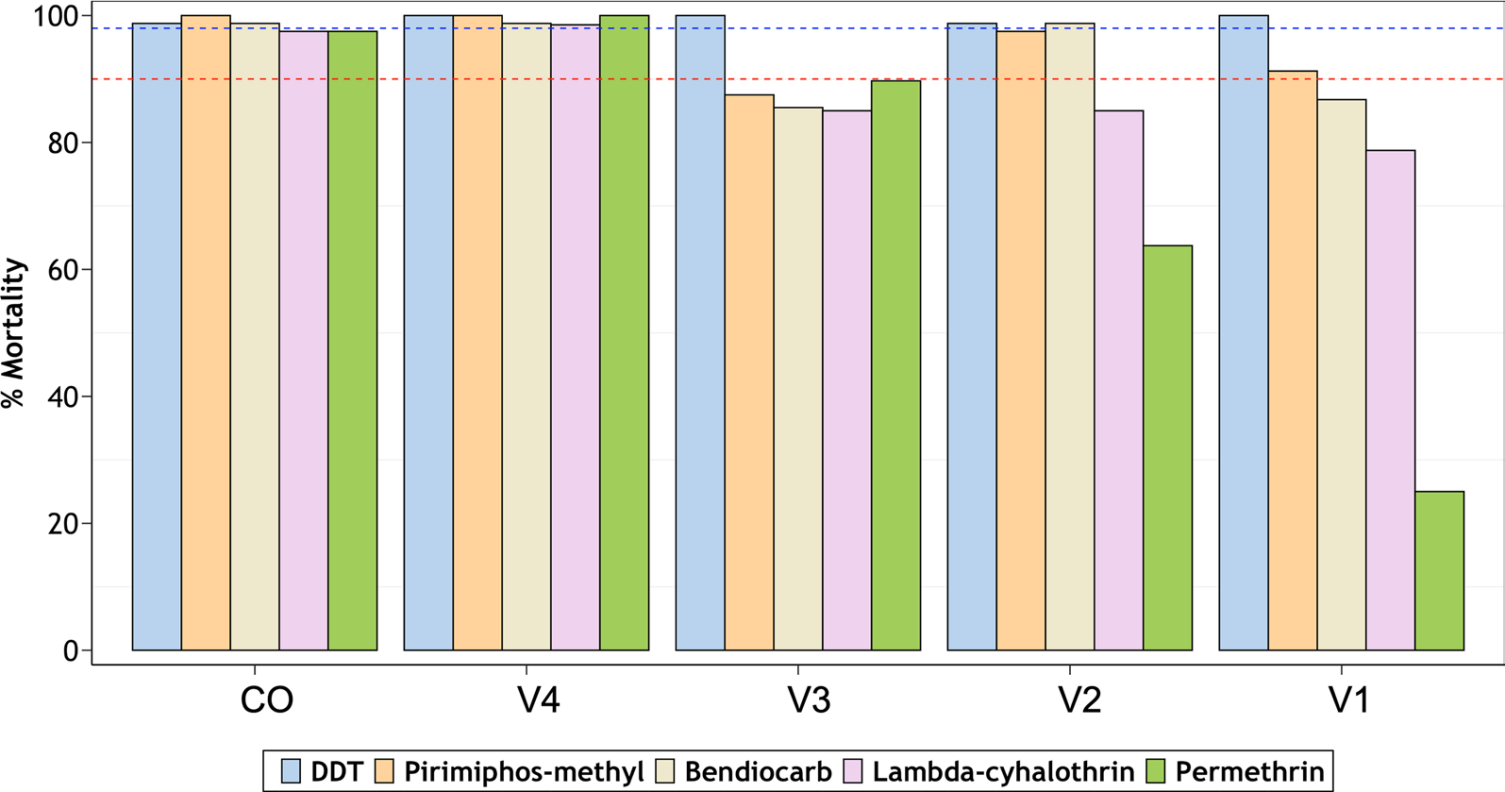
Percentage mortality in field-collected *Anopheles gambiae* (s.l.) exposed to standard concentrations of five insecticides. The red- and blue-dotted intercepts represent 90% and 98% mortalities indicative of resistance or susceptibility, respectively. Laboratory-reared mosquitoes are used as reference (CO = laboratory colony, V1 = Minepa, V2 = Lupiro, V3 = Kisawasawa, V4 = Njage)

The susceptibility test results are summarised in Fig. 2 and Table 3. *Anopheles arabiensis* from all four villages were susceptible to DDT. Resistance to the two candidate pyrethroids, lambda cyhalothrin and permethrin, was observed in three of the four villages (V1, V2 and V3), while resistance to the carbamate, bendiocarb was observed in two villages (V1 and V3). Similarly for the organophosphate pyrimiphos methyl, there was resistance in mosquitoes from V1 and V3 as well as signs of possible resistance in V2. Contrarily, mosquitoes from V4 were susceptible to all the candidate insecticides tested (mortality range: 98–100%) (Fig. 2). Comparative tests done using laboratory-reared mosquitoes of the same species revealed full susceptibility to all the candidate insecticides (Fig. 2). The knockdown times, including time taken to 50% knockdown, were recorded for the two pyrethroids and the organochloride and are summarised in Table 3.

**Table 3:** Knockdown time (KDT_50_) of *An. arabiensis* mosquitoes to three insecticide classes

	Organochloride			Pyrethroids					
	DDT			Permethrin			Lambda-cyhalothrin		
	KDT50	% Mortality	RR (95% CI)	KDT_50_	% Mortality	RR (95% CI)	KDT50	% Mortality	RR (95% CI)
Lab-reared	31.5 (30.0–32.9)	98.75	1	17.2 (16.0–18.5)	97.5	1	9.0 (6.4–11.0)	97.5	1
V1	43.9 (41.7–46.0)	100	1.4 (1.4–1.4)	37.1 (34.6–39.6)	25	1.9 (1.8–1.9)	38.0 (33.6–43.6)	78.75	4.2(4.0–5.2)
V2	35.5 (33.3–37.5)	98.75	1.13 (1.1–1.1)	28.5 (26.1–30.9)	63.75	1.6 (1.6–1.6)	17.3 (14.1–20.4)	85	1.9 (1.9–2.2)
V3	28.6 (26.4–30.9)	100	0.9 (0.9–0.9)	18.3 (16.8–19.8)	89.71	1.5 (1.4–1.5)	18.4 (15.2–21.4)	85	2.04 (1.9–2.4)
V4	26.5 (24.4–28.7)	100	0.8 (0.8–0.9)	21.5 (20.0–23.1)	100	1.3 (1.2–1.4)	19.4 (18.5–20.3)	98.5	2.2 (1.8–2.9)

V1 = Minepa, V2 = Lupiro, V3 = Kisawasawa, V4 = Njage

*DDT* dichloro-diphenyl-trichloroethane, *CI* confidence interval, *KDT*_*50*_ knockdown time (50%), *KDT90* knockdown time (90%), *RR* resistance ratio (KDT_50_ of exposed group ÷ KDT_50_ of the susceptible group)

#### Effects of the synergist, piperonyl butoxide (PBO), and possible involvement of metabolic resistance

**Fig. 3:**
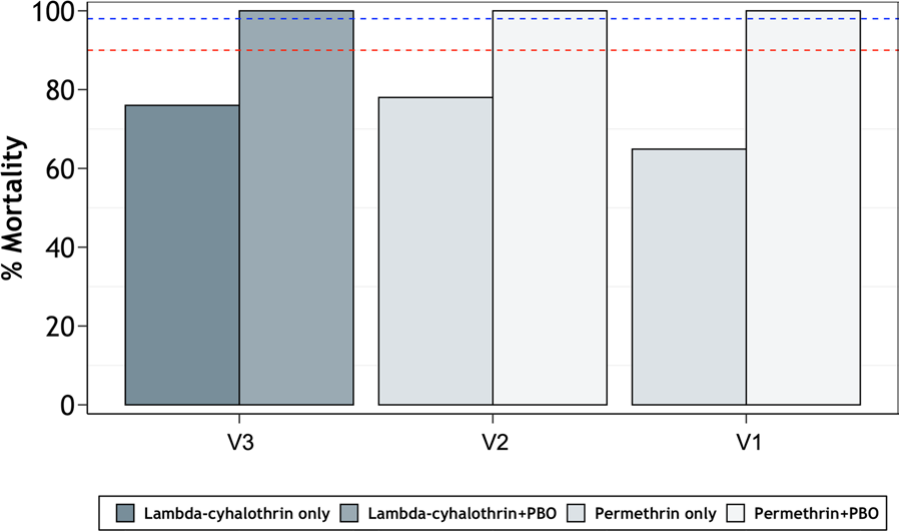
Percentage mortality of field collected *Anopheles gambiae* (s.l.) mosquitoes exposed to permethrin and lambda-cyhalothrin, with or without pre-exposure to piperonyl butoxide (PBO), in the three study villages (V1 = Minepa, V2 = Lupiro, V3 = Kisawasawa). The two dotted lines denote 98% and 90% mortality, respectively

In the three villages where there was pyrethroid resistance (V1–V3), the potency of the two pyrethroids was restored when mosquitoes were first exposed to the synergist, PBO, prior to the insecticide exposure. The resulting mortalities exceeded 98% in all cases across the three villages (Fig. 3).

#### Fecundity

**Fig. 4:**
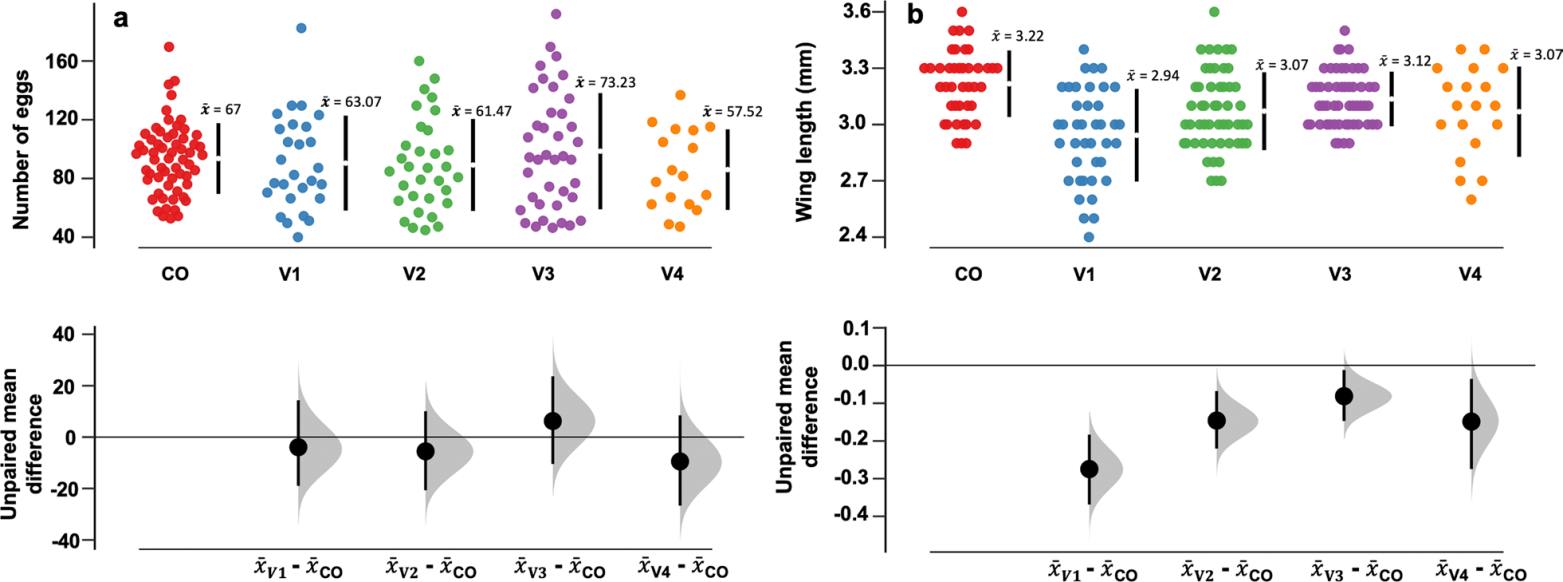
Fecundity estimates: **a** Estimated means of the number of eggs laid by field-collected *Anopheles gambiae* (s.l.) in four different villages (CO = colony, V1 = Minepa, V2 = Lupiro, V3 = Kisawasawa, V4 = Njage). **b** Estimated mean wing lengths of field *An. gambiae* (s.l.) in the four study villages. The data from laboratory-reared females are included as a reference. The vertical lines represent the 95% confidence levels, and the gap between the lines represents the mean. The black dots indicate mean differences relative to the reference group (in this case the number of eggs laid by laboratory-reared females). The filled gray curves indicate the resampled mean difference distribution of the number of eggs laid with reference to the colony unexposed mosquitos. The degree of significance is measured by how far the means of residuals varied from the reference line (0)

Mosquitoes collected from the field had clutch sizes with eggs ranging from 8 to 190 (V1), 6–150 (V2), 12–178 (V3) and 9–121 (V4) as estimated by direct counts under a dissecting microscope (Fig. 4a). The highest number of eggs laid was in V3, where the mean number of eggs was 73.23 (95% CI 55.7–88.7). The analysis of the mean residual differences showed that overall the fecundity was similar across the villages. There were also no significant differences in numbers of eggs laid by the field-collected relative to the laboratory-reared female mosquitoes.

#### Wing length

For field mosquitoes, the wing length ranged between 2.4 and 3.2 mm, with variations between the villages (Fig. 4b). V3 mosquitoes had the largest wings [mean wing length = 3.1 mm (95%  CI 3.06–3.14)] whereas V1 mosquitoes had the smallest wings [mean wing length = 2.9 mm (95% CI 2.82–2.98)]. In all study villages, the field-collected females were all smaller than females from the reference laboratory-reared colonies, which had wing lengths ranging from 2.6 mm to 3.6 mm (Fig. 4b).

### Effects of sub-lethal pesticide exposures on fecundity and wing lengths of mosquitoes

Following the initial tests, a lethal concentration (LC) of 15% mortality was chosen for subsequent experiments because it enabled sub-lethal exposure while also ensuring sufficient adult emergence (Table 4). The effects of the sub-lethal exposures on the levels of insecticide susceptibility, fecundity and wing lengths of emergent adults are summarised below.

**Table 4:** Sub-lethal concentrations used in laboratory pre-exposure experiments

Treatment	Active ingredient	Concentration	Dose (g/l)	95% CI (g/l)
Pyrethroid (Ninja 5EC)	Lambda-cyhalothrin	LC_15_	2.3 × 10^−7^	3.35 × 10^−6^−2.33 × 10^−7^
Organophosphate (Twigaphos)	Pirimiphos-methyl	LC_15_	1.0 × 10^−7^	9.42 × 10^−13^–6.36 × 10^−7^
Carbamate (Akheri)	Carbaryl	LC_15_	2.1 × 10^−4^	4.86 × 10^−3^–1.1 × 10^−4^

*CI* confidence interval

#### Insecticide susceptibility

**Fig. 5:**
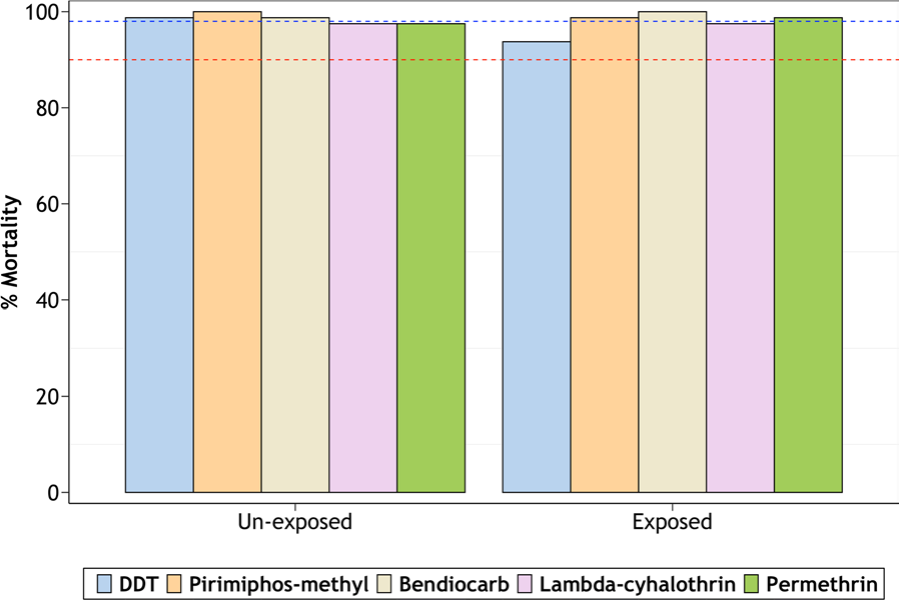
Percentage mortality in mosquitoes emerging from larvae that have been exposed or not exposed to insecticides. The mosquitoes were challenged with the same insecticides to which they had been pre-exposed

There were no clear differences in insecticide susceptibility between mosquitoes emerging from exposed and non-exposed larvae. However, when the larvae were pre-exposed to the pyrethroids and DDT, the emergent adults appeared slightly less susceptible to these same insecticides (Fig. 5).

#### Fecundity

**Fig. 6:**
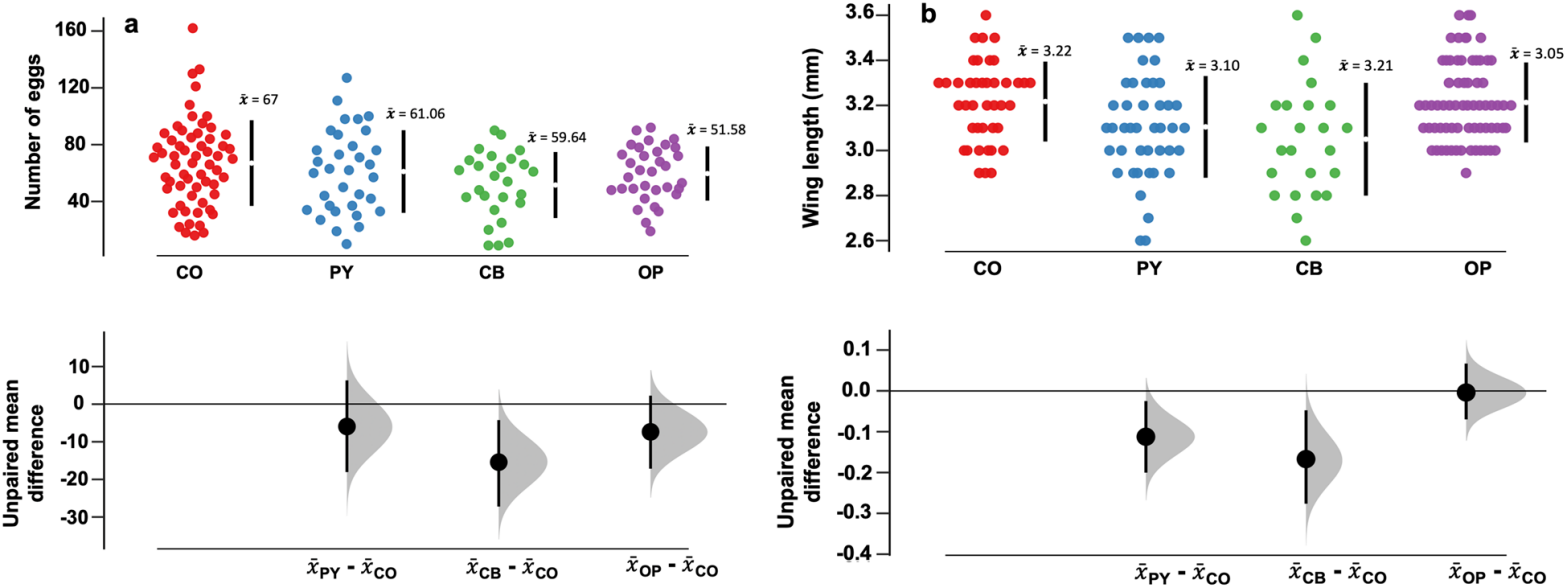
**a** Estimated mean number of eggs laid by *Anopheles arabiensis* mosquitoes emerging from larvae exposed to different pesticides; **b** estimated mean wing sizes of female *Anopheles arabiensis* mosquitoes emerging from larvae exposed to different pesticides. The specific pyrethroid was lambda-cyhalothrin, the carbamate was carbaryl and the organophosphate was pirimiphos-methyl. The degree of significance is measured by how far the means of the residuals varied from the reference line (0)

There were only marginal reductions in fecundity of laboratory-reared mosquitoes emerging from larvae exposed to pesticides, except in the case of organophosphate exposure, which significantly reduced fecundity. The un-exposed mosquitoes had egg clutches with a range of 16–162 eggs/female. In comparison, there were 19–92 eggs in carbamate-exposed mosquitoes, 10–127 eggs in pyrethroid-exposed mosquitoes and 9–90 eggs in organophosphate-exposed mosquitoes (Fig. 6a). The unexposed mosquitoes had the highest number of eggs [mean = 67 (95%  CI 59.4–74.6)] while those exposed to organophosphates had the lowest fecundity [mean = 52 (95% CI 43.1–60.1)].

#### Wing lengths

In the mosquitoes from pesticide-exposed larvae, wing length ranged between 2.6 mm and 3.6 mm, with the organophosphate group showing the lowest mean wing length. The unexposed group had the largest wings (Fig. 6b). Significant reductions of wing sizes were observed after exposure to pyrethroids and organophosphates. Contrarily, exposure to carbamates did not influence the wing lengths.

## Discussion

Insecticide resistance in malaria vectors might not be solely due to public health use in interventions, such as ITNs and IRS. For more consolidated insecticide resistance management, the agricultural sector must therefore be an integral part of malaria control and elimination programmes. The WHO Resistance Management Plan already borrows heavily from agricultural lessons but additional integration is necessary at the level of implementation [64]. To support this, research is needed to assess how disease vectors are affected in communities where pesticide exposures arise from the two sectors variedly. This study investigated potential impacts of agrochemicals on the fitness and susceptibility of malaria vectors to commonly used public health insecticides. The study relied on a combination of methods including focus group discussions and direct observations to explore agricultural practices in four villages, followed by direct experimental assessments of resistance, fecundity and wing lengths. Additional tests were conducted in which laboratory-reared mosquitoes were exposed to sub-lethal concentrations of pesticides and the same fitness parameters assessed compared to controls without the pre-exposures.

Previous studies have postulated that large-scale agrochemical use may increase resistance levels in malaria vector populations and therefore compromise the performance of public health tools such as ITNs and IRS [34, 35, 37, 38]. Separately, it has also been demonstrated that resistant mosquitoes incur certain fitness costs and may therefore not be as efficient vectors as susceptible ones [47–50]. However, a wider analysis of these factors suggests variations that make it difficult to draw a conclusion on the overall direction of the impacts [41]. It is therefore important that local studies are conducted to investigate the inter-linkages between agricultural practices and the responsiveness of malaria vectors to control by insecticidal interventions.

This study demonstrated that *An. arabiensis* mosquitoes in the study villages in south-eastern Tanzania were generally resistant to insecticides used in public health and agriculture, except in villages that reported minimal pesticide use. Though this work does not itself fully elucidate the role of agrochemicals in the development of insecticide resistance, the observations are indicative of a pathway of association, possibly even causal. The study village that had the highest levels of susceptibility was also the one with the lowest pesticide use as reported during the qualitative studies. Villages reporting pesticide use multiple times in a space of a week could perhaps create a highly insecticide-concentrated environments, resulting in heightened selection pressures. It remains unclear how much pesticide use is necessary to generate the negatively impactful selection pressures; thus, more research is required in this area. Since the chemical classes are common in both public health and agriculture, and because agriculture uses far higher pesticide quantities than public health, it is often assumed that resistance can arise in vector populations from either source. Evidently, studies have shown resistance to certain chemicals soon after introduction or even without actual public health use [65].

The qualitative findings of this study revealed a broad use of agrochemicals by farmers, often without appropriate guidance on dosage and waste disposal. This corroborates earlier studies conducted in the same area by Matowo et al. [16]. Additionally, a lack of awareness by farmers about the negative effects of agrochemicals on malaria control was noted and further emphasizes the need to provide targeted training and sensitization to improve pesticide stewardship in crop-farming communities. Fortunately, this study also revealed that the farmers were eager and willing to undergo formal training to better understand the link between the two sectors. It is necessary to urgently integrate practices in both sectors to ensure insecticide-resistance management strategies are effective.

Insecticide potency in pyrethroids (lambda-cyhalothrin and permethrin) was fully restored (to 100%) by first exposing resistant field mosquitoes to the synergist PBO. This restoration could be a sign of metabolic resistance as the mixed-function oxidase enzymes within these mosquitoes may have been suppressed [58]. This indicates that even if field mosquitoes are resistant to pyrethroids, bed nets impregnated with pyrethroids and PBO could be considered as a supplementary tool to the conventional LLINs [66]. Additionally, newer interventions such as non-pyrethroid actives along with non-pyrethroid IRS may result in greater community protection against malaria [67].

This study also showed that the fitness parameters such as fecundity and wing lengths only marginally reflected the differences in agricultural pesticide use. However, KDT_50_ for DDT and lambda-cyhalothrin increased profoundly in colony exposed mosquitoes. These data further confirm the observations that even low concentrations of insecticide exposure could alter the tolerance of mosquitoes to insecticides, and hence repetitive exposure across several generations might induce insecticide resistance [68]. Since these experiments were done with laboratory colonies and observations made on emergent adults, it is likely that the effects of sub-lethal pesticide exposure require multiple generations to manifest in the mosquito fitness. There were however clear signs that in some cases, especially when the pesticide exposure was from organophosphate and pyrethroids, the mosquitoes had significantly reduced fecundity and wing lengths in the emergent adults within the same generation. This could give an indication that induced resistance in mosquitoes could indirectly affect fecundity through a reduction in body size, or inversely, possibly suggesting that mosquito behavioural and biological responses may be pesticide specific as observed in Kibuthu et al. [69]. A similar observation was made for *An. funestus* in Tanzania whereby mosquitoes with small wing lengths laid fewer eggs compared to their larger counterparts (Nambunga et al. unpublished data). This is therefore an area of research requiring additional investigations to fully understand the causal pathways and the important factors.

Findings from this study showed that field mosquitoes from all four villages were significantly smaller compared to the colony unexposed group. This was an expected finding as various studies have shown that mosquitoes from the wild are much smaller than those reared in the laboratory [70–72]. This difference may be linked to the type of environment and resources available for the insect to thrive. Laboratory mosquitoes tend to have a more conducive environment, including nutritious food, minimal overcrowding, frequent blood meals, no insecticide selection pressure, and optimum temperature and humidity [70, 73, 74].

Even though the main objectives of this study were achieved, there were various limitations. First, field mosquito collections were done during the dry season; thus, the findings might be different during the wet season as shown by Matowo et al. [52]. Second, only one lethal concentration was used for sub-lethal exposure because of a limited number of colony mosquitoes. Third, no molecular analysis was done to identify the morphologically indistinguishable members of the *An. gambiae* complex since recent studies from the same area have indicated the complex now nearly exclusively comprises *An. arabiensis* [53]. Lastly, sub-lethal pesticide exposures were done only over a single generation and therefore did not allow for multi-generational observations of potential effects. It is recommended for future studies to perform susceptibility bioassays during both wet and dry seasons as well as use a range of lethal concentrations for sub-lethal dose experiments across multiple generations. Such studies should also consider other forms of pesticide use, such as on livestock, which may also influence insecticide susceptibility and fitness of the mosquitoes, especially where the vector species have zoophilic tendencies.

## Conclusion

For more consolidated insecticide resistance management plans, the agricultural sector must be integral in malaria control and elimination programmes. Moreover, safeguarding the potential of insecticide-based interventions requires improved understanding of how agricultural pesticides influence important life cycle processes and transmission potential of mosquito vectors. In this study, susceptibility of mosquitoes to public health insecticides was lower in villages reporting frequent use of pesticides compared to villages with little or no pesticide use. Variations in the fitness parameters, fecundity and wing length marginally reflected the differences in exposure to agricultural pesticides and should be investigated further. Pesticide use may impart additional life cycle constraints on mosquito vectors, but this is likely to occur over multiple-generational exposures.

## Data Availability

All data generated and supporting the conclusions of this manuscript have been included in this article. Raw data and material are available from the corresponding author upon request (NU).

## Abbreviations

IRS: Indoor residual spraying
IHI: Ifakara Health Institute
ITNs: Insecticide-treated nets

## References

[1] Bhatt S, Weiss DJ, Cameron E, Bisanzio D, Mappin B, Dalrymple U (2015). The effect of malaria control on *Plasmodium falciparum* in Africa between 2000 and 2015. Nature.

[2] Wilson AL, Courtenay O, Kelly-Hope LA, Scott TW, Takken W, Torr SJ (2020). The importance of vector control for the control and elimination of vector-borne diseases. PLoS Negl Trop Dis.

[3] Killeen GF (2014). Characterizing, controlling and eliminating residual malaria transmission. Malar J.

[4] Moyes CL, Athinya DK, Seethaler T, Battle KE, Sinka M, Hadi MP (2020). Evaluating insecticide resistance across African districts to aid malaria control decisions. Proc Natl Acad Sci USA.

[5] Kiszewski A, Mellinger A, Spielman A, Malaney P, Sachs SE, Sachs J (2004). A global index representing the stability of malaria transmission. Am J Trop Med Hyg.

[6] WHO (2021). World malaria report 2021.

[7] Mwangangi JM, Mbogo CM, Orindi BO, Muturi EJ, Midega JT, Nzovu J (2013). Shifts in malaria vector species. Malar J.

[8] Sougoufara S, Harry M, Doucouré S, Sembène PM, Sokhna C (2016). Shift in species composition in the *Anopheles gambiae* complex after implementation of long-lasting insecticidal nets in Dielmo, Senegal. Med Vet Entomol.

[9] Cooke MK, Kahindi SC, Oriango RM, Owaga C, Ayoma E, Mabuka D (2015). “A bite before bed”: exposure to malaria vectors outside the times of net use in the highlands of western Kenya. Malar J.

[10] Moiroux N, Damien GB, Egrot M, Djenontin A, Chandre F, Corbel V (2014). Human exposure to early morning *Anopheles funestus* biting behavior and personal protection provided by long-lasting insecticidal nets. PLoS ONE.

[11] Hemingway J, Hawkes NJ, McCarroll L, Ranson H (2004). The molecular basis of insecticide resistance in mosquitoes. Insect Biochem Mol Biol.

[12] Ijumba JN, Mosha FW, Lindsay SW (2002). Malaria transmission risk variations derived from different agricultural practices in an irrigated area of northern Tanzania. Med Vet Entomol.

[13] Hawkes C, Ruel M (2006). The links between agriculture and health: An intersectoral opportunity to improve the health and livelihoods of the poor. Bull World Health Organ.

[14] Mutero CM, Mccartney M, Boelee E (2006). Understanding the links between agriculture health. Vis Focus.

[15] Janko MM, Irish SR, Reich BJ, Peterson M, Doctor SM, Mwandagalirwa MK (2018). The links between agriculture, *Anopheles* mosquitoes, and malaria risk in children younger than 5 years in the Democratic Republic of the Congo: a population-based, cross-sectional, spatial study. Lancet Planet Heal.

[16] Matowo NS, Tanner M, Munhenga G, Mapua SA, Finda M, Utzinger J (2020). Patterns of pesticide usage in agriculture in rural Tanzania call for integrating agricultural and public health practices in managing insecticide-resistance in malaria vectors. Malar J.

[17] Philbert A, Lyantagaye SL, Nkwengulila G (2019). Farmers’ pesticide usage practices in the malaria endemic region of North-Western Tanzania: implications to the control of malaria vectors. BMC Public Health.

[18] Rulisa A, Van KL (2022). When local trade-offs between SDGs turn out to be wealth-dependent : interaction between expanding rice cultivation and eradicating malaria in Rwanda. Sustainability.

[19] Paul P, Kangalawe RYM, Mboera LEG (2018). Land-use patterns and their implication on malaria transmission in Kilosa District, Tanzania. Trop Dis Travel Med Vaccines.

[20] Lipton M, De Kadt E, WHO (1988). Agriculture-health linkages.

[21] Ijumba JN, Lindsay SW (2001). Impact of irrigation on malaria in Africa: paddies paradox. Med Vet Entomol.

[22] Mwangangi JM, Shililu J, Muturi EJ, Muriu S, Jacob B, Kabiru EW (2010). *Anopheles* larval abundance and diversity in three rice agro-village complexes Mwea irrigation scheme, central Kenya. Malar J.

[23] Oguoma V, Ikpeze O (2009). Species composition and abundance of mosquitoes of a tropical irrigation ecosystem. Anim Res Int.

[24] Demissew A, Hawaria D, Kibret S, Animut A, Tsegaye A, Lee MC (2020). Impact of sugarcane irrigation on malaria vector *Anopheles* mosquito fauna, abundance and seasonality in Arjo-Didessa, Ethiopia. Malar J.

[25] Lacey LA, Lacey CM (1990). The medical importance of riceland mosquitoes and their control using alternatives to chemical insecticides. J Am Mosq Control Assoc Suppl.

[26] Bradley DJ (1988). The epidemiology of ricefield-associated diseases. Vector-borne dis control humans through rice agroecosystem Manag.

[27] Chan K, Tusting LS, Bottomley C, Saito K, Djouaka R, Lines PJ (2022). Malaria transmission and prevalence in rice-growing versus non-rice-growing villages in Africa: a systematic review and meta-analysis. Lancet Planet Heal.

[28] Mwangangi JM, Muturi EJ, Shililu J, Muriu SM, Jacob B, Kabiru EW (2006). Survival of immature Anopheles arabiensis (Diptera: Culicidae) in aquatic habitats in Mwea rice irrigation scheme, central Kenya. Malar J.

[29] Mukiama TK, Mwangi RW (1989). Field studies of larval *Anopheles*
*arabiensis* patton of Mwea irrigation scheme, Kenya. Int J Trop Insect Sci.

[30] Takken W, Verhulst NO (2013). Host preferences of blood-feeding mosquitoes. Annu Rev Entomol.

[31] Killeen GF, McKenzie FE, Foy BD, Bøgh C, Beier JC (2001). The availability of potential hosts as a determinant of feeding behaviours and malaria transmission by African mosquito populations. Trans R Soc Trop Med Hyg.

[32] Kisinza W, Kabula B, Tungu P, Sindato C, Mweya C, Massue D (2011). Detection and monitoring of insecticide resistance in malaria vectors in Tanzania Mainland.

[33] N’Guessan R, Corbel V, Akogbéto M, Rowland M (2007). Reduced efficacy of insecticide-treated nets and indoor residual spraying for malaria control in pyrethroid resistance area, Benin. Emerg Infect Dis.

[34] Ranson H, Abdallah H, Badolo A, Guelbeogo WM, Kerah-Hinzoumbé C, Yangalbé-Kalnoné E (2009). Insecticide resistance in *Anopheles gambiae*: data from the first year of a multi-country study highlight the extent of the problem. Malar J.

[35] Matowo J, Kulkarni MA, Mosha FW, Oxborough RM, Kitau JA, Tenu F (2010). Biochemical basis of permethrin resistance in *Anopheles arabiensis* from Lower Moshi, north-eastern Tanzania. Malar J.

[36] Nkya TE, Akhouayri I, Kisinza W, David JP (2013). Impact of environment on mosquito response to pyrethroid insecticides: facts, evidences and prospects. Insect Biochem Mol Biol.

[37] Overgaard H (2006). Malaria mosquito resistance to agricultural insecticides: risk area mapping in Thailand.

[38] Yadouleton A, Martin T, Padonou G, Chandre F, Asidi A, Djogbenou L (2011). Cotton pest management practices and the selection of pyrethroid resistance in *Anopheles gambiae* population in northern Benin. Parasit Vectors.

[39] Fodjo BK, Koudou BG, Tia E, Saric J, N’Dri PB, Zoh MG (2018). Insecticide resistance status of *An*. *gambiae* in areas of varying agrochemical use in Côte D’Ivoire. Biomed Res Int.

[40] Vanek MJ, Shoo B, Mtasiwa D, Kiama M, Lindsay SW, Fillinger U (2006). Community-based surveillance of malaria vector larval habitats: a baseline study in urban Dar es Salaam, Tanzania. BMC Public Health.

[41] Freeman JC, Smith LB, Silva JJ, Fan Y, Sun H, Scott JG (2021). Fitness studies of insecticide resistant strains: lessons learned and future directions. Pest Manag Sci.

[42] Chen TY, Smartt CT, Shin D (2021). Permethrin resistance in *Aedes aegypti* affects aspects of vectorial capacity. Insects.

[43] Tabachnick W (1994). Genetics of insect vector competence for arboviruses, Advances in disease vector research.

[44] Beerntsen BT, James AA, Christensen BM (2000). Genetics of mosquito vector competence. Microbiol Mol Biol Rev.

[45] William C, Black IV, Bennett KE, Gorrochotegui-Escalante N, Barillas-Mury CV, Fernandez-Salas I, de LourdesMunoz M (2002). Flavivirus susceptibility in *Aedes aegypti*. Arch Med Res.

[46] Brito LP, Linss JGB, Lima-Camara TN, Belinato TA, Peixoto AA, Lima JBP (2013). Assessing the Effects of *Aedes aegypti* kdr mutations on pyrethroid resistance and its fitness cost. PLoS ONE.

[47] Antonio-Nkondjio C, Nkahe DL, Kopya E, Djiappi-Tchamen B, Toussile W, Sonhafouo-Chiana N (2020). Fitness cost of insecticide resistance on the life-traits of a *Anopheles coluzzii* population from the city of Yaoundé, Cameroon. Wellcome Open Res.

[48] Osoro JK, Machani MG, Ochomo E, Wanjala C, Omukunda E, Munga S (2021). Insecticide resistance exerts significant fitness costs in immature stages of *Anopheles gambiae* in western Kenya. Malar J.

[49] Collins E, Vaselli NM, Sylla M, Beavogui AH, Orsborne J, Lawrence G (2019). The relationship between insecticide resistance, mosquito age and malaria prevalence in *Anopheles*
*gambiae *s.l. from Guinea. Sci Rep.

[50] Smith LB, Silva JJ, Chen C, Harrington LC, Scott JG (2021). Fitness costs of individual and combined pyrethroid resistance mechanisms, Kdr and CYP-mediated detoxification, in *Aedes aegypti*. PLoS Negl Trop Dis.

[51] David MR, Garcia GA, Valle D, Maciel-De-Freitas R (2018). Insecticide resistance and fitness: the case of four *Aedes aegypti* populations from different Brazilian regions. Biomed Res Int.

[52] Matowo NS, Munhenga G, Tanner M, Coetzee M, Feringa WF, Ngowo HS (2017). Fine-scale spatial and temporal heterogeneities in insecticide resistance profiles of the malaria vector, *Anopheles arabiensis* in rural south-eastern Tanzania. Wellcome Open Res..

[53] Pinda PG, Eichenberger C, Ngowo HS, Msaky DS, Abbasi S, Kihonda J (2020). Comparative assessment of insecticide resistance phenotypes in two major malaria vectors, *Anopheles funestus* and *Anopheles arabiensis* in south-eastern Tanzania. Malar J.

[54] Kaindoa EW, Matowo NS, Ngowo HS, Mkandawile G, Mmbando A, Finda M (2017). Interventions that effectively target *Anopheles funestus* mosquitoes could significantly improve control of persistent malaria transmission in south-eastern Tanzania. PLoS ONE.

[55] World Weather Online. Morogoro monthly climate averages [Internet]. 2021. Available from: https://www.worldweatheronline.com/morogoro-weather-averages/morogoro/tz.aspx.

[56] Kato F (2007). Development of a major rice cultivation area in the Kilombero Valley, Tanzania. African Study Monogr.

[57] Renggli S, Mandike R, Kramer K, Patrick F, Brown NJ, McElroy PD (2013). Design, implementation and evaluation of a national campaign to deliver 18 million free long-lasting insecticidal nets to uncovered sleeping spaces in Tanzania. Malar J.

[58] WHO (2016). Test procedures for insecticide resistance monitoring in malaria vector mosquitoes.

[59] Team RC. A language and environment for statistical computing [Internet]. R Packag. version. 2021. Available from: https://cran.r-project.org/src/base/R-4/

[60] Wheeler MW, Park RM, Bailer AJ (2006). Comparing median lethal concentration values using confidence interval overlap or ratio tests. Environ Toxicol Chem.

[61] Chang W. R graphics cookbook. Beijing: O’Reily; 2013.

[62] Software L. POLO-Plus 1.0 Probit and Logit Analysis. Petaluma: LeOra Software; 2006.

[63] Ho J, Tumkaya T, Aryal S, Choi H, Claridge-Chang A (2019). Moving beyond P values: data analysis with estimation graphics. Nat Methods.

[64] WHO (2012). Global Plan for Insecticide Resistance Managment.

[65] Ranson H (2017). Current and future prospects for preventing malaria transmission via the use of insecticides. Cold Spring Harb Perspect Med.

[66] Asidi A, N’Guessan R, Akogbeto M, Curtis C, Rowland M (2012). Loss of household protection from use of insecticide-treated nets against pyrethroid-resistant mosquitoes, Benin. Emerg Infect Dis.

[67] Protopopoff N, Mosha JF, Lukole E, Charlwood JD, Wright A, Mwalimu CD (2018). Effectiveness of a long-lasting piperonyl butoxide-treated insecticidal net and indoor residual spray interventions, separately and together, against malaria transmitted by pyrethroid-resistant mosquitoes: a cluster, randomised controlled, two-by-two fact. Lancet.

[68] Nkya TE, Mosha FW, Magesa SM, Kisinza WN (2014). Increased tolerance of *Anopheles gambiae *s.s. to chemical insecticides after exposure to agrochemical mixture. Tanzan J Health Res.

[69] Kibuthu TW, Njenga SM, Mbugua AK, Muturi EJ (2016). Agricultural chemicals: Life changer for mosquito vectors in agricultural landscapes?. Parasit Vectors.

[70] Yeap HL, Endersby NM, Johnson PH, Ritchie SA, Hoffmann AA (2013). Body size and wing shape measurements as quality indicators of *Aedes aegypti* mosquitoes destined for field release. Am J Trop Med Hyg.

[71] Briegel H, Knüsel I, Timmermann S (2001). *Aedes aegypti*: size, reserves, survival, and flight potential. J Vector Ecol.

[72] Mogi M, Miyagi I, Abadi K, Syafruddin.  (1996). Inter- and Intraspecific Variation in Resistance to Desiccation by Adult *Aedes* (*Stegomyia*) spp. (Diptera: *Culicidae*) from Indonesia. J Med Entomol.

[73] Leemingsawat S, Dujardin J-P (2008). The geometry of the wing of *Aedes* (*Stegomyia*) aegypti in isofemale lines through successive generations. Infect Genet Evol.

[74] Jirakanjanakit N, Dujardin J, Pathom N (2005). Discrimination of *Aedes*
*aegypti* (Dipteria *Culicidae*) laboratory lines based on wing geometry. Southeast Asian J Trop Med Public Health.

